# Immobilization of a Bifidobacterial Endo-*ß-N*-Acetylglucosaminidase to Generate Bioactive Compounds for Food Industry

**DOI:** 10.3389/fbioe.2022.922423

**Published:** 2022-07-22

**Authors:** Burcu Pekdemir, Hatice Duman, Ayşenur Arslan, Merve Kaplan, Melda Karyelioğlu, Tolgahan Özer, Hacı Mehmet Kayılı, Bekir Salih, Bethany M. Henrick, Rebbeca M. Duar, Sercan Karav

**Affiliations:** ^1^ Department of Molecular Biology and Genetics, Canakkale Onsekiz Mart University, Canakkale, Turkey; ^2^ Department of Biomedical Engineering, Karabuk University, Karabuk, Turkey; ^3^ Department of Chemistry, Hacettepe University, Ankara, Turkey; ^4^ Department of Food Science and Technology, University of Nebraska Lincoln, Lincoln, NE, United States; ^5^ Evolve BioSystems Inc. Davis, Davis, CA, United States

**Keywords:** *B. infantis*, Endo-*ß-N*-acetylglucosaminidase, *N*-glycans, immobilization, bioactive compounds

## Abstract

Conjugated *N*-glycans are considered next-generation bioactive prebiotic compounds due to their selective stimulation of beneficial microbes. These compounds are glycosidically attached to proteins through *N*-acetylglucosamines *via* specific asparagine residue (AsN-X-Ser/Thr). Certain bacteria such as *Bifidobacterium longum subspecies infantis* (*B. infantis*) have been shown to be capable of utilizing conjugated *N*-glycans, owing to their specialized genomic abilities. *B. infantis* possess a unique enzyme, Endo-*ß-N*-acetylglucosaminidase (EndoBI-1)**,** which cleaves all types of conjugated *N*-glycans from glycoproteins. In this study, recombinantly cloned EndoBI-1 enzyme activity was investigated using various immobilization methods: 1) adsorption, 2) entrapment-based alginate immobilization, 3) SulfoLink-, and 4) AminoLink-based covalent bonding immobilization techniques were compared to develop the optimum application of EndoBI-1 to food processes. The yield of enzyme immobilization and the activity of each immobilized enzyme by different approaches were investigated. The *N*-glycans released from lactoperoxidase (LPO) using different immobilized enzyme forms were characterized using MALDI-TOF mass spectrometry (MS). As expected, regardless of the techniques, the enzyme activity decreased after the immobilization methods. The enzyme activity of adsorption and entrapment-based alginate immobilization was found to be 71.55% ± 0.6 and 20.32% ± 3.18, respectively, whereas the activity of AminoLink- and SulfoLink-based covalent bonding immobilization was found to be 58.05 ± 1.98 and 47.49% ± 0.30 compared to the free form of the enzyme, respectively. However, extended incubation time recovery achieved activity similar to that of the free form. More importantly, each immobilization method resulted in the same glycan profile containing 11 different *N*-glycan structures from a model glycoprotein LPO based on MALDI-TOF MS analysis. The glycan data analysis suggests that immobilization of EndoBI-1 is not affecting the enzyme specificity, which enables full glycan release without a limitation. Hence, different immobilization methods investigated in this study can be chosen for effective enzyme immobilization to obtain bioactive glycans. These findings highlight that further optimization of these methods can be a promising approach for future processing scale-up and commercialization of EndoBI-1 and similar enzymes.

## 1 Introduction

Bioactive compounds are defined as nutrient or non-nutrient substances which can have physiological, behavioral, and immunological effects (Biesalski et al., 2009). These compounds are extensively studied to reveal their potential antioxidant, anti-inflammatory, and antimicrobial activities with various *in vitro* and *in vivo* studies. Bioactive compounds are derived from both plant sources, including phenolics and carotenoids, and animal sources containing milk peptide and fatty acids (Patil et al., 2009; Arslan et al., 2021). Novel bioactive compounds are used in the fields of functional foods, pharmaceuticals, nutrition, biotechnology, and agriculture, which results in increased numbers of nutritional products with a myriad of health benefits (Biesalski et al., 2009; Kaplan et al., 2022). To date, *N-*glycans are regarded as a bioactive compound with scientific and technological developments (Karav et al., 2015b; Karav, 2018). In nature, glycans are oligosaccharides that can be covalently attached to protein structures by *O*-glycosidic or *N*-glycosidic bonds (Stanley et al., 2009). *O*-linked glycans (*O*-glycans) are mostly linked to a polypeptide chain at a serine/threonine residue and can be extended into several core classes, while *N*-linked glycans (*N*-glycans) are linked through *N*-acetylglucosamines (HexNAc) to a specific asparagine residue (AsN-X-Ser/Thr). The *N*-glycan core consists of two HexNAc residues followed by three mannoses, and the *N*-glycan types are determined through further glycosylation with other monosaccharides. *N*-glycans are basically classified into three main classes including high mannose (HM), complex type (CT), and hybrid (HY) based on the degree of branching and the type of linkages (Ohtsubo and Marth, 2006; Stanley et al., 2009). As with the other important macromolecules, *N*-glycans are one of the most significant bioactive compounds that show a variety of biological activities including cell adhesion and mobility, receptor activation, protein folding and degradation, immune signaling, and protection against pathogens (Barboza et al., 2012; Duman et al., 2021). In addition, *N*-glycans have prebiotic properties since they are indigestible by human digestive enzymes, which means they can reach the colon intact and be utilized by certain bacterial species found in the gut microbiota. Thus, these compounds can serve as novel prebiotic substrates that can selectively stimulate the growth of specific beneficial bacteria such as Bifidobacteria (Koropatkin et al., 2012; Karav et al., 2019). Furthermore, as *N*-glycans are similar to human milk oligosaccharides (HMOs) in terms of the monosaccharide composition and linkage, they are proposed to have a bifidogenic effect (Karav et al., 2016). These bioactive compounds enhance the growth of Bifidobacteria and in turn shape the gut microbiota. Another noticeable effect of *N*-glycans is that they are fermented to short-chain fatty acids (SCFAs) such as acetate and lactate, which creates an unfavorable environment for the growth of pathogens. Consequently, bioactive *N*-glycans provide colonization resistance to pathogens and reduce inflammation as well as virulence factors (Duar et al., 2020).

The release of *N*-glycans from glycoproteins is a crucial step in both experimentally determining biological characteristics and the large-scale production of these bioactive molecules (Helenius, 2001). *N*-glycans can be released from glycoproteins using different chemical and enzymatic treatments, but the current deglycosylation methods require harsh treatment and extreme reaction conditions such as pH or temperature (Karav et al., 2017). Although chemical treatments including beta-elimination, hydrazinolysis, and trifluoromethanesulfonic acid are considered as easy-to-use, low cost, and rapid, they can degrade the glycan structures, limiting further analysis of these glycans (Tams and Welinder, 1998; Nakakita et al., 2007; Zauner et al., 2012). Peptide-N4-(N-acetyl-b-D-glucosaminyl) asparagine amidases (PNGases) is a type of glycosidase, a group of enzymes commonly used for releasing *N*-glycans from glycoprotein moieties (Takahashi, 1977; Nuck et al., 1990). Similar to chemical methods, enzymatic deglycosylation treatments also have various limitations. To increase the enzyme accessibility to the glycans, the enzymatic reaction with PNGases requires high temperatures and different detergents for the deglycosylation process. This might result in the degradation of the intact form of glycans and remaining polypeptide chains (Tarentino and Plummer, 1994; Fryksdale et al., 2002). While other commercial endoglycosidases such as endoglycosidases H (Endo H) and endoglycosidases F1, F2, and F3 (Endo F1, F2, and F3) show more activity for the deglycosylation of native glycans from glycoproteins when compared to PNGases, these endoglycosidases show low activity on the deglycosylation of multiple-antennary types *N*-glycans (Trimble and Tarentino, 1991; Garrido et al., 2012). Therefore, the development of an effective deglycosylation method for releasing *N*-glycans without any degradation of their intact form is warranted (Karav, 2019; Sucu et al., 2021). Endo-*β-N*-acetylglucosaminidase (EndoBI-1) is a novel enzyme that is recently isolated from *Bifidobacterium longum subspecies infantis* (*B. infantis*) ATCC 15697 (Garrido et al., 2012; Karav et al., 2015b). This novel enzyme cleaves *N-N*’diacetyl chitobiose moiety found in all types of *N*-glycans, and core fucosylation has no effect on the enzyme activity. It is also considered as heat resistant and shows activity during 95°C heat treatments (Karav et al., 2015a; Karav et al., 2015b; Parc et al., 2015). These properties make EndoBI-1 ideal for several applications, especially for the food industry (Karav et al., 2015b; Parc et al., 2015; Şahutoğlu et al., 2020; Duman et al., 2021).

Enzymes can be unsuitable for direct application to different industrial processes because of their biological origin, activity, and different reaction conditions including pH and temperature. (Yamaguchi et al., 2020). In such cases, they have limited stability, and also do not possess an optimum catalytic feature. Thus, different immobilization approaches can help overcome these drawbacks (Garcia-Galan et al., 2011; Sheldon and van Pelt, 2013; Yamaguchi et al., 2020). In general, the process of immobilization is the attachment or adsorption of a soluble enzyme to solid support, the entrapment in a matrix, or the aggregation of the enzyme, allowing enzyme reuse during production (Cao et al., 2003; Datta et al., 2013). Several enzyme immobilization methods have been improved and used since Nelson and Griffin in 1916. Enzyme immobilization methods can be classified as reversible immobilization and irreversible immobilization methods. While the reversible immobilization method is further grouped as adsorption, ionic binding, affinity binding, and metal binding, the irreversible immobilization method is grouped as covalent bonding, entrapment, and aggregation (Karav et al., 2017). Generally, immobilization techniques can have some drawbacks. For example, reversible immobilization techniques can cause enzyme leaching that prevents enzyme reusability or irreversible enzyme immobilization may minimize enzyme activity because of the active site blockage and inherent diffusion problems (Karav et al., 2017). Thus, the optimal immobilization method for the enzyme is determined according to the physicochemical properties of the enzyme, support material, and substrate matrix that are used in the immobilization process (Nisha et al., 2012).

In this study, different types of immobilization strategies including adsorption, entrapment, and covalent bonding such as AminoLink- and SulfoLink-based covalent bonding methods were evaluated for the potential application of EndoBI-1 in the food industry in terms of enzyme activity and specificity.

## 2 Materials and Methods

### 2.1 Reagents, Enzymes, and Substrates

The Sulfolink™ Immobilization Kit (44995), the AminoLink™ Immobilization Kit (44890), and Qubit™ Protein Assay Kit (Q33211) were purchased from Thermo Fisher Scientific (Waltham, MA Unites States). Calcium chloride (CaCl_2_), sodium alginate (Na-alginate), sodium chloride (NaCl), sodium phosphate dibasic dihydrate (Na_2_HPO_4_·2H_2_O), sodium phosphate monobasic (NaH_2_PO_4_), and lactoperoxidase (LPO) from bovine milk were obtained from Sigma Aldrich Chemical Co. (St Louis, Missouri, United States).

### 2.2 Recombinant Enzyme Production

An Expresso^®^ Rhamnose Cloning & Protein Expression *in vivo* cloning system (Lucigen Corp., Middleton, WI, United States) was preferred for gene cloning, and the manufacturer’s protocols were followed. The coding sequence of EndoBI-1 (Blon_2468) was amplified from *B. infantis* ATCC 15697 genomic DNA using appropriate cloning primers and then cloned into the pRham™ N-His Kan vector. The enzyme was cloned by adding an N-terminal poly-histidine tag (His-tag) without transmembrane domains and signal peptide sequences to facilitate protein expression and purification. The vector was transformed into *Escherichia cloni* (*E. cloni*) 10G chemically competent cells (Lucigen Corp.). After the confirmation of positive clones using Colony PCR, *E. cloni* 10G cells containing the vector were incubated at 37°C, and 2.5–3 h (h) duration to reach the optical density at 600 nm of ∼0.6. Protein expression was induced by the addition of rhamnose solution (0.2% final concentration). The enzyme was purified after the bacterial lysis process using affinity chromatography with the Ni-charged columns (Bio-Rad, Hercules, CA, United States). The expression and purity of the enzyme were confirmed by 12–4% SDS-PAGE gel electrophoresis. The recombinantly produced EndoBI-1 was kept at −80°C for further analysis (Supplemental Figure S1, S2).

### 2.3 Enzyme Immobilization

#### 2.3.1 Enzyme Immobilization by Adsorption Method

**FIGURE 1:**
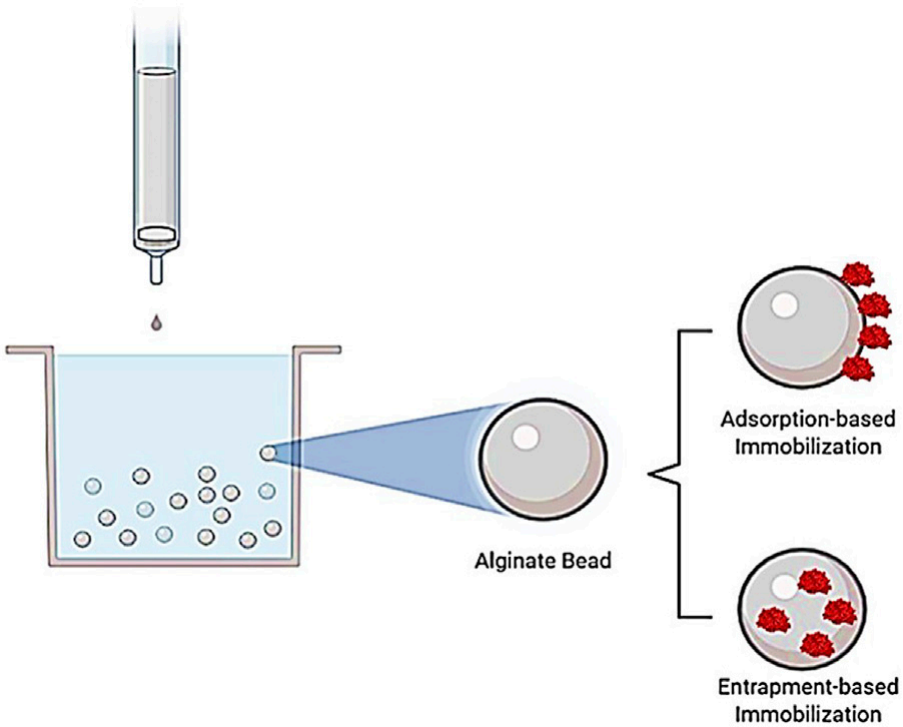
Representation of adsorption- and entrapment-based immobilization methods. In the adsorption-based immobilization method, the soluble enzyme attaches to solid support, whereas the soluble enzyme is entrapped in a support material in the entrapment-based immobilization method.

For the immobilization of EndoBI-1 using the adsorption method, Na-alginate was selected as a support material (Figure 1). To achieve the highest immobilization efficiency, different pH levels of sodium acetate solution were used during the immobilization process. A total of 1 g of Na-alginate powder was dissolved in 50 ml of dH_2_O to prepare 2% Na-alginate solution, and 1.25 g of CaCl_2_ powder was dissolved in 25 ml of dH_2_O to prepare 5% CaCl_2_ buffer solution. The Na-alginate solution was then dripped into a 5% CaCl_2_ solution through a G22 syringe to form alginate beads. The alginate beads were filtered through filter paper and washed with dH_2_O. For the enzyme adsorption, 0.1 M sodium acetate solutions at different pH (6.34, 4.83, and 3.63) were mixed with EndoBI-1 (1.88 mg/ml) and alginate beads were added to each treatment while mixing. Each mixture was stirred for 1–1.30 h. The concentration of alginate-bound and unbound enzyme was determined fluorometrically two times using the Qubit protein assay kit.

#### 2.3.2 Enzyme Immobilization by Entrapment Method

For the immobilization of EndoBI-1 using the entrapment method, Na-alginate was used as a support material (Figure 1). The alginate bead concentration is the most important criteria for the entrapment type immobilization; therefore, different concentrations of Na-alginate solution (2%, 3%, and 4%) were used to investigate the immobilization efficiency. First, EndoBI-1 (1.88 mg/ml) was added into each Na-alginate solution. The mixtures obtained were extruded dropwise using a G22 syringe, into a gently stirred 5% CaCl_2_ solution. The formed beads were left in the CaCl_2_ solution with a stirrer for about 10 min (min). After washing with dH_2_O of beads, the Qubit assay kit was used for the measurement of nonentrapped and entrapped enzyme concentrations**.** The measurement was performed two times.

#### 2.3.3 Enzyme Immobilization by SulfoLink Covalent Bonding Method

**FIGURE 2:**
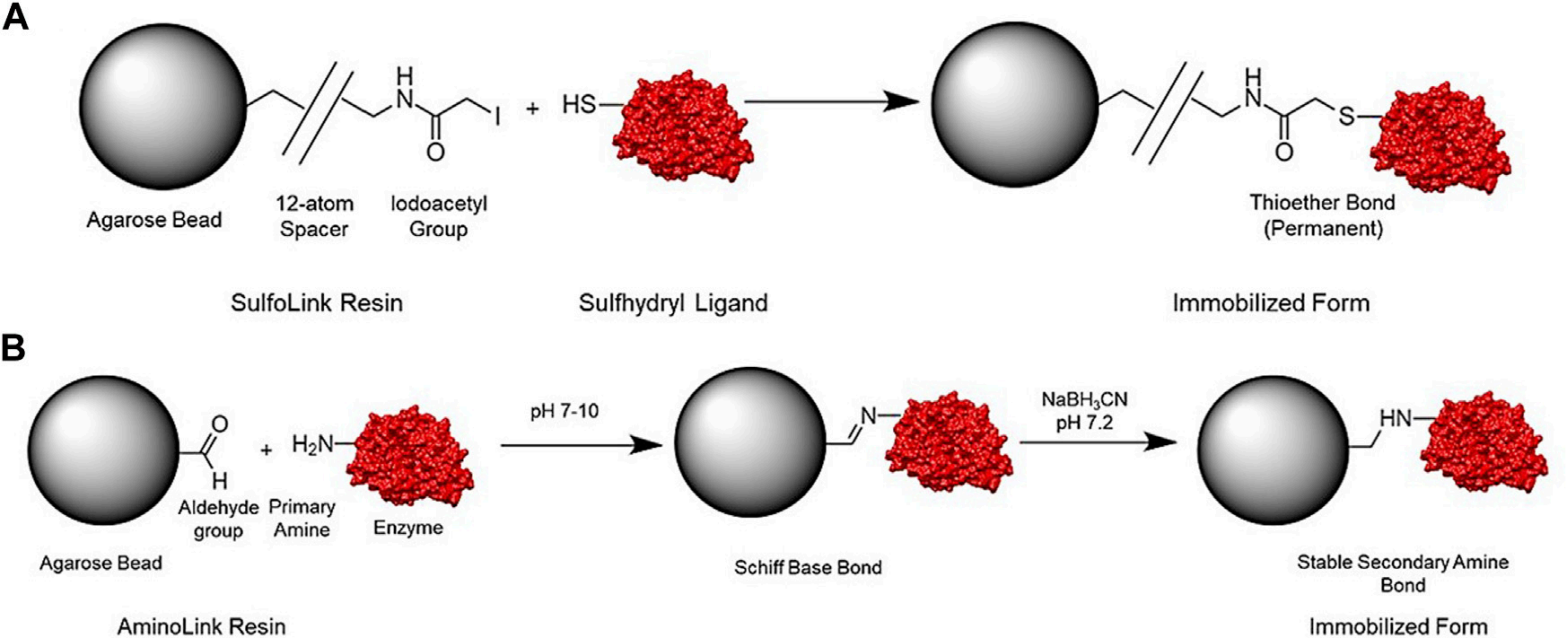
Representation of general structure and reaction scheme for the SulfoLink **(A)** and AminoLink coupling resin **(B)**.

EndoBI-1 was immobilized on the SulfoLink coupling resin employing the SulfoLink Immobilization Trial Kit (Thermo Scientific), following the manufacturer’s directions (Figure 2). To prepare the enzyme for coupling, 1.88 mg/ml of EndoBI-1 was dissolved in 1 ml of sample preparation buffer, and the enzyme solution was transferred to the vial containing 6 mg of 2-mercaptoethylamine•HCl (2-MEA) (50 mM) and incubated for 1.5 h at 37°C. The desalt spin column which is provided with the kit was equilibrated using the coupling buffer and centrifuged at 1,000 x *g* for 1 min at 25°C. After this process was applied one more time, the enzyme solution was injected into the column containing the compact resin bed, and 0.1 ml of coupling buffer was added into the column. The reduced enzyme was collected after centrifugation at 1,000 x *g* for 1 min at 25°C for later determining coupling efficiency. For SulfoLink-based immobilization, SulfoLink resin was suspended gently by end-over-end mixing and centrifuged at 1,000 x *g* for 1 min at 25°C to remove the storage buffer from the column. A total of 2 ml of coupling buffer was added into the column, and the centrifugation process was applied at 1,000 x *g* for 1 min at 25°C. The sulfhydryl-containing enzyme solution was transferred to the SulfoLink column, and the column was incubated at room temperature for 15 min with end-over-end mixing and then for 30 min without mixing. After incubation, the SulfoLink column was placed into a new tube and centrifuged at 1,000 x *g* for 1 min at 25°C to collect nonbound enzyme. Finally, the column was rinsed with wash solution and was then stored at 4°C based on the manufacturer’s protocol. Immobilized enzyme concentration was measured two times using the Qubit protein assay kit.

#### 2.3.4 Enzyme Immobilization by AminoLink Covalent Bonding Method

EndoBI-1 was immobilized on the AminoLink coupling resin using the AminoLink Plus Immobilization Trial Kit (Thermo Scientific) according to the manufacturer’s directions (Figure 2). For AminoLink-based immobilization, the coupling buffer solution and storage buffer solution were prepared according to the manufacturer’s instructions using the kit contents. To prepare the enzyme solution, 4 ml of pH 10.0 coupling buffer solution was mixed with 1 ml of enzyme solution (1.88 mg/ml) completely. The AminoLink plus resin was suspended by end-over-end mixing, and the column was centrifuged in a 15 ml falcon tube at 1,000 x *g* for 1 min at 24°C to remove the storage buffer. A total of 2 ml of pH 10.0 coupling buffer was added into the column, and the column was then centrifuged at 1,000 x *g* for 1 min at 24°C. After this process was applied one more time, the enzyme solution was injected into the column containing the compact resin bed, and 0.1 ml of coupling buffer was added into the column. A total of 0.1 ml of the sample was stored at 4°C for the measurement of coupling efficiency later. After this, the column was incubated for 2 h by end-over-end rocking and incubated for 2 h while kept upright without mixing at room temperature. After incubation, the caps of the column were removed and placed into a new tube. To collect nonbound enzymes, centrifugation was carried out at 1,000 x *g* for 1 min at 24°C. The column was washed with 2 ml of pH 7.2 phosphate-buffered saline (PBS) solution and centrifuged. A total of 2 ml of pH 7.2 coupling buffer was mixed with 40 μL of sodium cyanoborohydride solution, and then the resulting solution was transferred to the column. Incubation at room temperature was conducted for the column by mixing for 4 h. Finally, the column was stored at 4°C according to the manufacturer’s directions. Immobilized enzyme concentration was measured fluorometrically two times using the Qubit protein assay kit.

### 2.4 Determination of Kinetic Parameters Using Lactoperoxidase

The kinetic parameters (*K*_*m*_ and *V*_*max*_) of free and immobilized EndoBI-1 by each method (adsorption, entrapment, SulfoLink-, and AminoLink-based covalent bonding) were evaluated by the measurement of enzyme activity on LPO as a substrate. The concentration of released glycans was determined using the phenol-sulfuric acid assay, calorimetrically. The kinetic parameters of free and immobilized EndoBI-1 were calculated using GraphPad Prism 9.0 (GraphPad Software Inc., San Diego). Data were analyzed and plotted by fitting to linear regression analysis (Lineweaver Burk) with the initial rates at different substrate concentrations (0.1–0.8 mg/ml and the incubation time of 20 min) based on the Michaelis–Menten equation. The kinetic parameters of free and immobilized EndoBI-1 were determined two times, and one-way ANOVA variance analysis along with Tukey’s multiple comparisons statistical test was performed to assess the statistical significance of the data at *p* < 0.05 using NCSS 12 statistical software.

### 2.5 Characterization of Released *N*-Glycans Using Matrix-Assisted Laser Desorption-Ionization-Time of Flight Mass Spectrometry

#### 2.5.1 Deglycosylation of Lactoperoxidase

To characterize the released *N*-glycans from LPO, first, 5 mg/ml LPO was deglycosylated using free and immobilized forms of EndoBI-1 enzyme (0.025 mg/ml) in 0.02 M Na_2_HPO_4_ buffer solution under optimized conditions (37°C, pH 5, and overnight). After the reaction was terminated and the released glycans were collected by protein precipitation using cold ethanol into the sample (4:1 v/v), and the substrate and enzyme concentrations were determined using the Qubit protein assay kit based on the manufacturer’s instructions.

#### 2.5.2 Characterization of *N*-Glycans

After enzymatic digestion and protein precipitation, *N*-glycans were characterized using a RapifleX MALDI Tissuetyper MS (Bruker Daltonics, Bremen, GmbH). The *N*-glycans released using different EndoBI-1 containing materials were labeled with 2-aminobenzoic acid (2-AA) as described previously with minor modifications (Ruhaak et al., 2008). A total of 50 μL of released *N*-glycan samples were mixed with 25 μL of 2-AA [48 mg/ml containing 15% (v/v) acetic acid in dimethyl sulfoxide (DMSO)] and 25 μL of 2-picoline-borane (Pic-BH_3_; 107 mg/ml in DMSO). The reaction solution was then incubated at 65°C for 2 h. After the labeling process, a cotton-containing pipette tip was used for the purification of *N*-glycans. The cotton fibers were placed on a 100-µL pipette tip. The 2-AA labeled samples (25 μL) were mixed with 140 μL of acetonitrile (ACN) to reach an optimal loading solution. The cotton-containing pipette tip was washed sequentially with 100 μL of dH_2_O and 100 μL of 85% ACN. The loading solution was then passed through the cotton-containing pipette tip at least 20 times. After that, the pipette tip was washed three times with ACN/dH_2_O/trifluoroacetic acid (TCA) solution (85/14/1 v/v/v), followed by 85% ACN to completely purify *N*-glycans from salt and other solvent residues. Elution was performed with 20 μL of dH_2_O prior to mass spectrometry analysis. A total of 1 μL of elution solution was dropped onto the MTP 384 anchor plate and dried. A total of 1 μL of DHB matrix (2-5 dihydroxy benzoic acid) prepared in ACN/dH_2_O (1:1 v/v) was then added to this spot. After crystallization, the analysis was performed by collecting at least 8,000 laser shots in reflectron mode in negative ionization. The Bruker RapifleX MALDI Tissuetyper MS was calibrated with a peptide mixture by applying 25 kV acceleration voltage, and mass spectra were acquired in the 1,000–4,000 mass range. The released *N*-glycan peaks obtained from MALDI-MS analysis were annotated by matching the theoretically obtained 2-AA–labeled *N*-glycan mass peaks that were generated by GlycoWorkbench software.

## 3 Results

### 3.1 Enzyme Immobilization Yield

**FIGURE 3:**
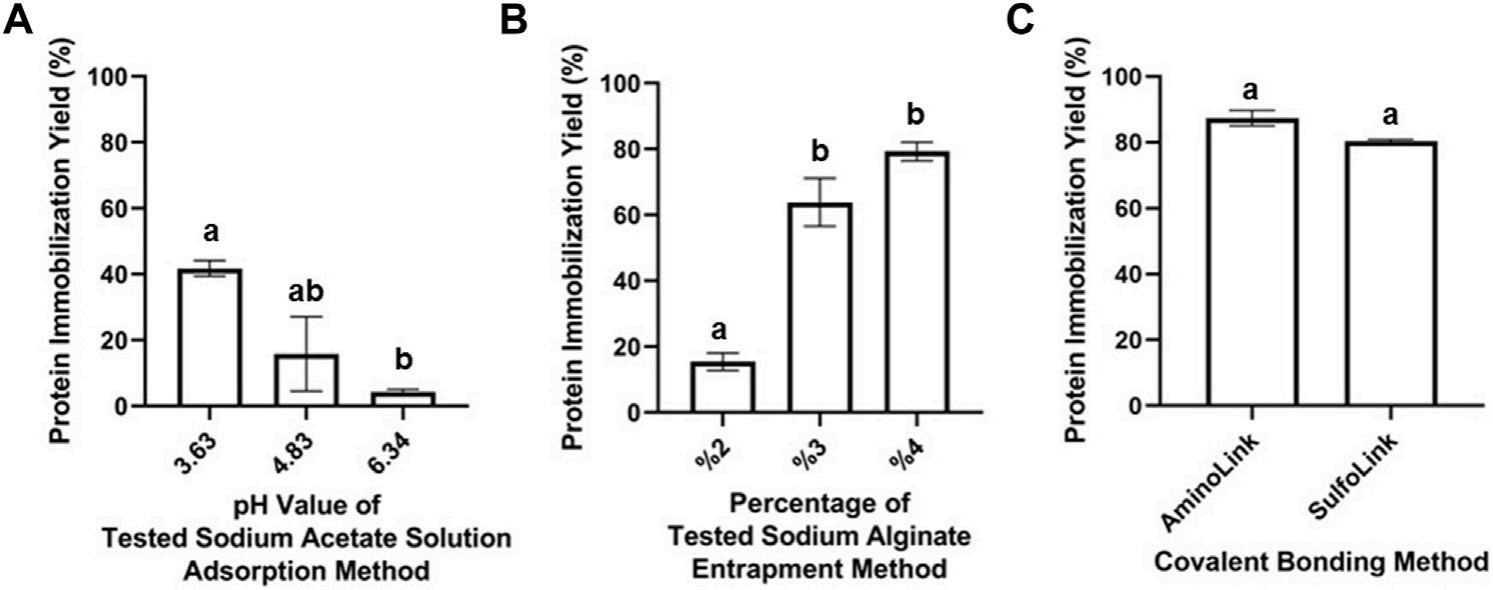
Protein immobilization yield for adsorption- **(A)**, entrapment- **(B)**, AminoLink- **(C)**, and SulfoLink-based **(C)** immobilization methods, measured fluorometrically by the Qubit Protein Assay Kit. Error bars represent standard deviation and mean values within the same graph, when followed by different letters (a,b, ab), they are significantly different at *p <* 0.05.

The enzyme immobilization yield of SulfoLink- and AminoLink-based covalent bonding, adsorption, and entrapment-immobilized EndoBI-1 was determined by quantifying the total protein and remaining unbound protein after the washing process. The enzyme immobilization yield was calculated using the following equation:

$$\text{Immobilization}\;\text{Yield}\;\left(\text{\%}\right)\;=\;\frac{Pt-Pu}{Pt}\;x\;100,$$

where *Pt* is the concentration of total protein and *Pu* is the remaining unbound protein concentration. All the measurements were carried out fluorometrically in triplicate by using the Qubit protein assay kit. The results of the fluorescence method demonstrated the high immobilization yield (87.41%) of the AminoLink-based covalent bonding method, whereas the SulfoLink-based covalent method immobilized 80.41% of the enzyme. Immobilization with the adsorption method resulted in the lowest immobilization yield. To investigate the highest immobilization efficiency, different pH levels of sodium acetate solution were tested during the immobilization process. Accordingly, the adsorption yield was found to be 4.28% at 6.34 pH, 15.83% at 4.83 pH, and 41.72% at 3.63 pH. The immobilization yield of the entrapment method was found to be 15.43, 63.83, and 79.26% for the different alginate bead concentrations (2, 3, and 4%, respectively) (Figure 3).

### 3.2 Kinetic Characterization Using Lactoperoxidase

Kinetic parameters of the free and immobilized EndoBI-1 enzyme on LPO with different techniques were determined according to the Michaelis–Menten model. First, preliminary trials were performed to determine the conditions where the [E] << [S] condition is fulfilled. The concentration of the glycan released from the protein substrate using constant enzyme concentrations at different time intervals and different substrate concentrations were determined. The glycans released from 0.1—0.8 mg/ml LPO by the free-form enzyme were observed for 50 min at 5-min intervals, and it was observed that the release of glycan increased linearly during the first 20 min for all concentrations, where the kinetic studies were carried out by calculating the slope for each concentration.

The kinetic parameters (*K*_*m*_ *, V*_*max*_) were calculated using the linearized Lineweaver–Burk plotting technique to reduce the error of nonlinear calculation. According to the analysis results, *K*_*m*_ values (3.51 ± 0.02, 6.16 ± 0.12, 3.18 ± 0.13, 2.69 ± 0.09, and 3.58 ± 0.02 mg/ml) and *V*_max_ values (6.87, 6.36, 2.09, 2.84, and 4.09 μg/mLxmin) were determined for the free form of EndoBI-1, and the immobilized enzyme forms including adsorption and entrapment-based alginate immobilization, SulfoLink-, and AminoLink-based covalent bonding immobilization, respectively.

**FIGURE 4:**
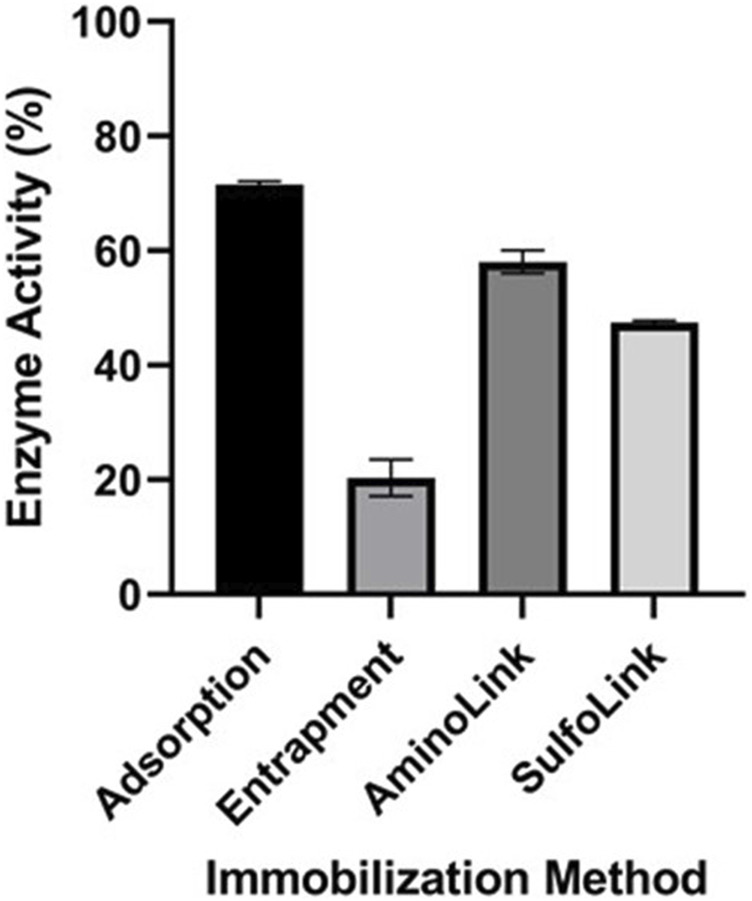
Enzyme activity of immobilized EndoBI-1 (adsorption, entrapment, AminoLink, and SulfoLink) on LPO. Error bars represent standard deviation and mean values of different groups and are significantly different at *p <* 0.05.

The *V*_*max*_ value from kinetic characterization analysis and the activity of free EndoBI-1 were used to estimate the immobilized enzyme activity. Adsorption on alginate beads immobilization had the highest activity with the entrapment, AminoLink-, and SulfoLink-based immobilization methods retaining 71.55, 20.32, 58.05, and 47.49% activity, respectively (Figure 4).

### 3.3 Characterization of Released *N*-Glycans Using Matrix-Assisted Laser Desorption-Ionization-Time of Flight Mass Spectrometry

**FIGURE 5:**
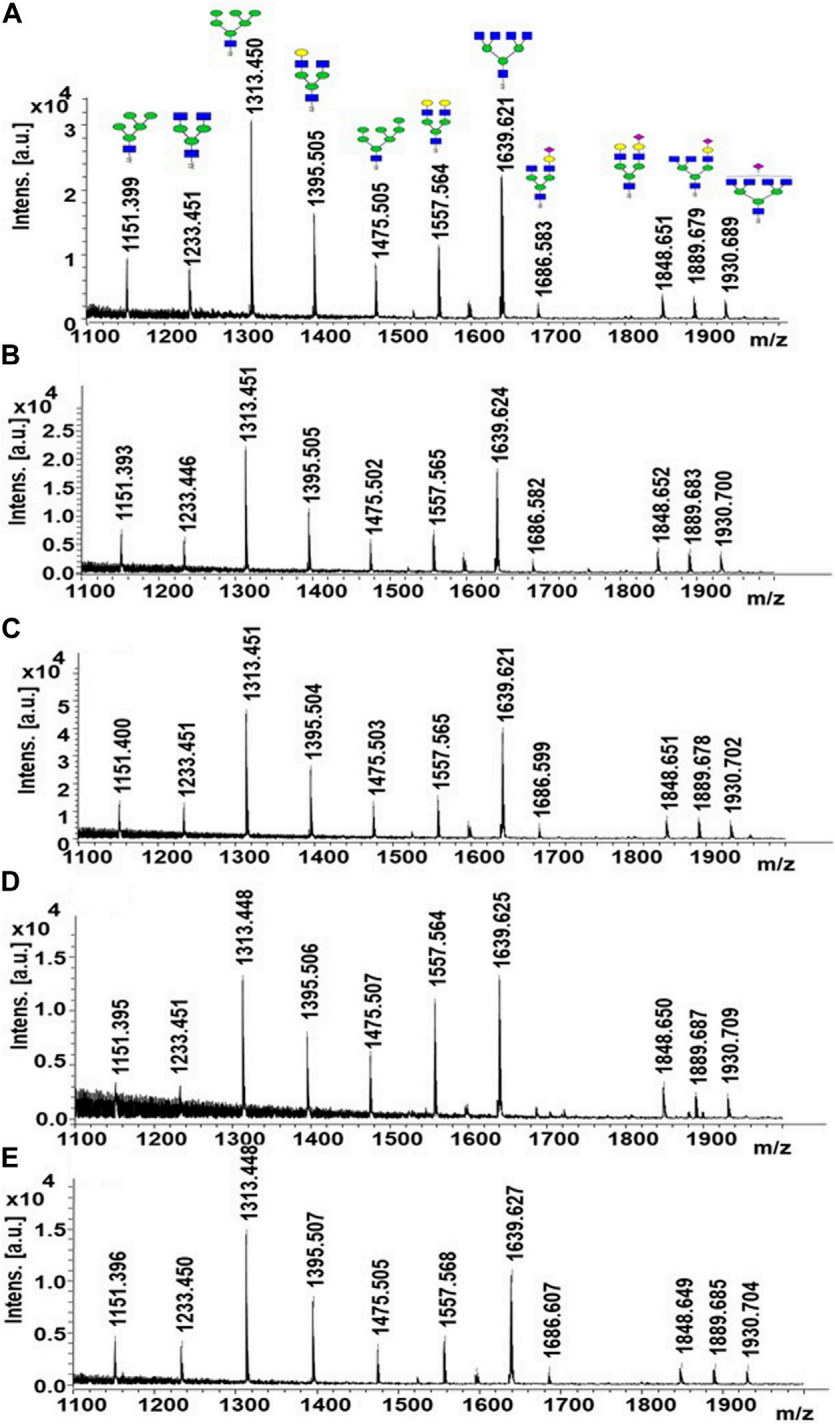
MALDI-MS spectra of released *N*-glycans obtained from free **(A)**, adsorption- **(B)**, entrapment- **(C)**, AminoLink- **(D)**, and SulfoLink-based **(E)** immobilized EndoBI-1.

The glycan compositions released from the LPO protein substrate by different enzyme forms are given in Table 1. In the study, bovine LPO was used as a standard glycoprotein to test the release of *N*-glycans using EndoBI-1 enzyme–containing materials. After the incubation of the samples with free EndoBI-1 enzyme and EndoBI-1 enzyme–containing materials, the released *N*-glycans were labeled with 2-AA, and analyzed by MALDI-MS. Figure 5 shows the MALDI mass spectra belonging to the detected *N*-glycans released from different immobilization approaches. According to the results, it was seen that all the enzymes immobilized by different immobilization techniques compared to the enzyme in free form (Figure 5A) could release the same glycans. These techniques showed different levels of activity in the enzyme kinetics study. However, when long-term incubation was provided, all enzyme forms were able to release all *N*-glycans. These results also showed that all immobilization techniques provide sufficient *N*-glycan release for the characterization of *N*-glycans by MS.

**TABLE 1:** The *N*-glycan compositions released from bovine LPO by EndoBI-1.

*N*-glycan composition	Experimental *m/z*	Theoretical *m/z*	Error (Da)
H5N1	1151.399	1151.400	0.001
H3N3	1233.451	1233.453	0.002
H6N1	1313.450	1313.452	0.002
H4N3	1395.505	1395.506	0.000
H7N1	1475.505	1475.505	0.000
H5N3	1557.564	1557.558	-0.006
H3N5	1639.621	1639.611	-0.010
H4N3S1	1686.583	1686.601	0.018
H5N3S1	1848.651	1848.654	0.003
H4N4S1	1889.679	1889.680	0.001
H3N5S1	1930.689	1930.707	0.018

## 4 Discussion

Enzymes are specialized catalysts for several chemical reactions and always play an important role in industrial processes, especially for food technology. Considering industrial processing, enzymes may be unsuitable for different applications because of their characteristic features such as biological origin, activity, and different conditions including pH and temperature. In such cases, they have limited stability, and also do not possess an optimum catalytic feature. Immobilization is an important strategy to overcome these limitations and improve enzyme activity, stability, reusability, and specificities.

In this study, EndoBI-1 enzyme was immobilized with different types of immobilization strategies including adsorption, entrapment, and covalent bonding such as AminoLink- and SulfoLink-based covalent methods. The protein immobilization yield and the activity of the enzyme immobilized by each method are investigated. MALDI-TOF MS analysis was used to characterize the *N*-glycans released from LPO using the different immobilized enzyme forms.

The immobilization yield of SulfoLink-, AminoLink-based covalent bonding, adsorption, and entrapment-immobilized EndoBI-1 was determined by quantifying the total protein and remaining unbound protein fluorometrically. Differences between immobilization yields have been observed because of their interaction specificities (hydrophobic interactions and covalent bonds). High binding efficiency was observed in the AminoLink-based immobilization method followed by SulfoLink-based immobilization and entrapment immobilization (4% alginate concentration). However, immobilization with the adsorption method resulted in the lowest immobilization yield compared to other immobilization techniques. This is an expected situation due to the weak binding forces between the enzyme molecules and the support material. This wear interaction can cause the leaching of enzymes from the support material, which hinders the reusability of the enzyme. It was also observed that the immobilization yield increased by decreasing the acidity level of sodium acetate solution in the adsorption method. In the entrapment method, the concentration of alginate beads is the most important criterion. As expected, the high immobilization yield was obtained from the high alginate bead concentration.

Considering the kinetic parameters of free and immobilized EndoBI-1, *K*_*m*_ and *V*_*max*_ values were calculated using the linearized Lineweaver–Burk plotting technique to reduce the error of nonlinear calculation. Optimized concentrations and levels of pH were used for the determination of kinetic parameters. According to the results, the goodness-of-fit for the linear regression analysis (R^2^) for all types of EndoBI-1 forms was found to be ≥ 0.99. *K*_*m*_ values for the entrapment and AminoLink-based immobilization methods were found to be lower than the free enzyme, whereas high *K*_*m*_ values were obtained after adsorption and SulfoLink-based immobilization when compared to the free form. Thus, these results showed that immobilized enzymes with adsorption and the SulfoLink-based covalent bonding method had low affinity for its substrate compared to the other forms. Typically, *V*_*max*_ values were found to be lower than the free enzyme. Based on these results, the adsorption on alginate beads immobilization had the most activity, while the least activity was observed in the entrapment immobilization method because of EndoBI-1 as a large substrate molecule that causes transfer problems.

MS analysis showed that all enzymes immobilized by different immobilization techniques compared to the enzyme in the free form can release the same types of glycans. These techniques showed different levels of activity in the enzyme kinetics study. However, extended incubation time overcame this limitation, resulting in the release of all *N*-glycans equivalent to the free form of the enzyme.

In conclusion, the activity of EndoBI-1, as preserved with all the immobilization methods tested in this study and with minor adjustments, and the activity of the immobilized enzymes matched that of the free form. These findings highlight that different immobilization approaches can be used for the effective enzyme immobilization without any limitations to obtain bioactive glycans. Thus, further optimization of these immobilization techniques can be a promising tool for the future processing scale-up and commercialization of EndoBI-1 and similar enzymes, along with obtaining bioactive glycans for industrial applications, particularly the food industry.

## Data Availability

The original contributions presented in the study are included in the article/Supplementary Materials. Further inquiries can be directed to the corresponding author.
