# Supplementary material for: Immobilization of a Bifidobacterial Endo-ß-N-Acetylglucosaminidase to Generate Bioactive Compounds for Food Industry
Source: Front Bioeng Biotechnol. 2022 Jul 22;10:922423. doi: 10.3389/fbioe.2022.922423 (PMC9353140; doi:10.3389/fbioe.2022.922423)
Supplement: Supplementary file 1 [file DataSheet2.PDF]

Supplementary Material

1.1 Supplementary Figures

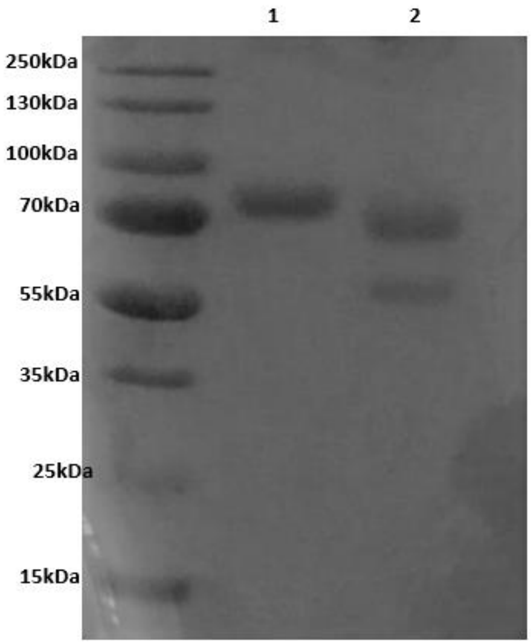

Supplementary Figure 2. Enzymatic deglycosylation of Lactoperoxidase (LPO) EndoBI-1 on 4-12% SDS-PAGE gel. Lane 1: Glycosylated LPO (78 kDa). Lane 2: LPO deglycosylated by EndoBI-1.
