# Supplementary material for: Immobilization of a Bifidobacterial Endo-ß-N-Acetylglucosaminidase to Generate Bioactive Compounds for Food Industry
Source: Front Bioeng Biotechnol. 2022 Jul 22;10:922423. doi: 10.3389/fbioe.2022.922423 (PMC9353140; doi:10.3389/fbioe.2022.922423)
Supplement: Supplementary file 2 [file DataSheet1.PDF]

Supplementary Material

1.1 Supplementary Figures

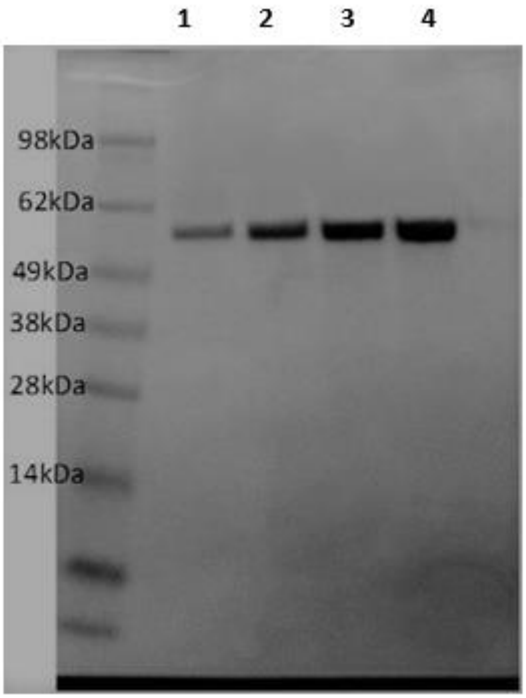

Supplementary Figure 1. SDS-PAGE gel of recombinant EndoBI-1. Lane 1: EndoBI-1 (2 µL); Lane 2: EndoBI-1 (5 µL); Lane 3: EndoBI-1 (10 µL); Lane 4: EndoBI-1 (20 µL).
